# Expanding the molecular-ruler process through vapor deposition of hexadecanethiol

**DOI:** 10.3762/bjnano.8.233

**Published:** 2017-11-07

**Authors:** Alexandra M Patron, Timothy S Hooker, Daniel F Santavicca, Corey P Causey, Thomas J Mullen

**Affiliations:** 1 Department of Chemistry, University of North Florida, Jacksonville, FL 32224, USA; 2 Department of Physics, University of North Florida, Jacksonville, FL 32224, USA

**Keywords:** hybrid nanolithography, metal-ligated multilayer, molecular ruler, self-assembled monolayers, vapor-phase deposition

## Abstract

The development of methods to produce nanoscale features with tailored chemical functionalities is fundamental for applications such as nanoelectronics and sensor fabrication. The molecular-ruler process shows great utility for this purpose as it combines top-down lithography for the creation of complex architectures over large areas in conjunction with molecular self-assembly, which enables precise control over the physical and chemical properties of small local features. The molecular-ruler process, which most commonly uses mercaptoalkanoic acids and metal ions to generate metal-ligated multilayers, can be employed to produce registered nanogaps between metal features. Expansion of this methodology to include molecules with other chemical functionalities could greatly expand the overall versatility, and thus the utility, of this process. Herein, we explore the use of alkanethiol molecules as the terminating layer of metal-ligated multilayers. During this study, it was discovered that the solution deposition of alkanethiol molecules resulted in low overall surface coverage with features that varied in height. Because features with varied heights are not conducive to the production of uniform nanogaps via the molecular-ruler process, the vapor-phase deposition of alkanethiol molecules was explored. Unlike the solution-phase deposition, alkanethiol islands produced by vapor-phase deposition exhibited markedly higher surface coverages of uniform heights. To illustrate the applicability of this method, metal-ligated multilayers, both with and without an alkanethiol capping layer, were utilized to create nanogaps between Au features using the molecular-ruler process.

## Findings

In a time when many technological advances are driven by the miniaturization of fabrication methods, much effort has been placed on the development of novel methods to produce nanoscale features with chemical functionalities that go beyond traditional semiconductors [1–3]. Recent advances in the field allow for the fabrication of molecular-scale features into surfaces that template the assembly and growth of metals, polymers, biomolecules, and cellular structures [3–11]. In addition, these surface assemblies have been utilized as molecular-scale resists for lithography [12–13]. One promising strategy for such fabrication utilizes top-down lithography to create complex architectures over large areas in conjunction with molecular self-assembly, which enables precise control over the physical and chemical properties of the small features [1–2]. The molecular-ruler process is a notable example of this hybrid approach as it couples conventional patterning methods with molecular self-assembly [14].

**Figure 1:**
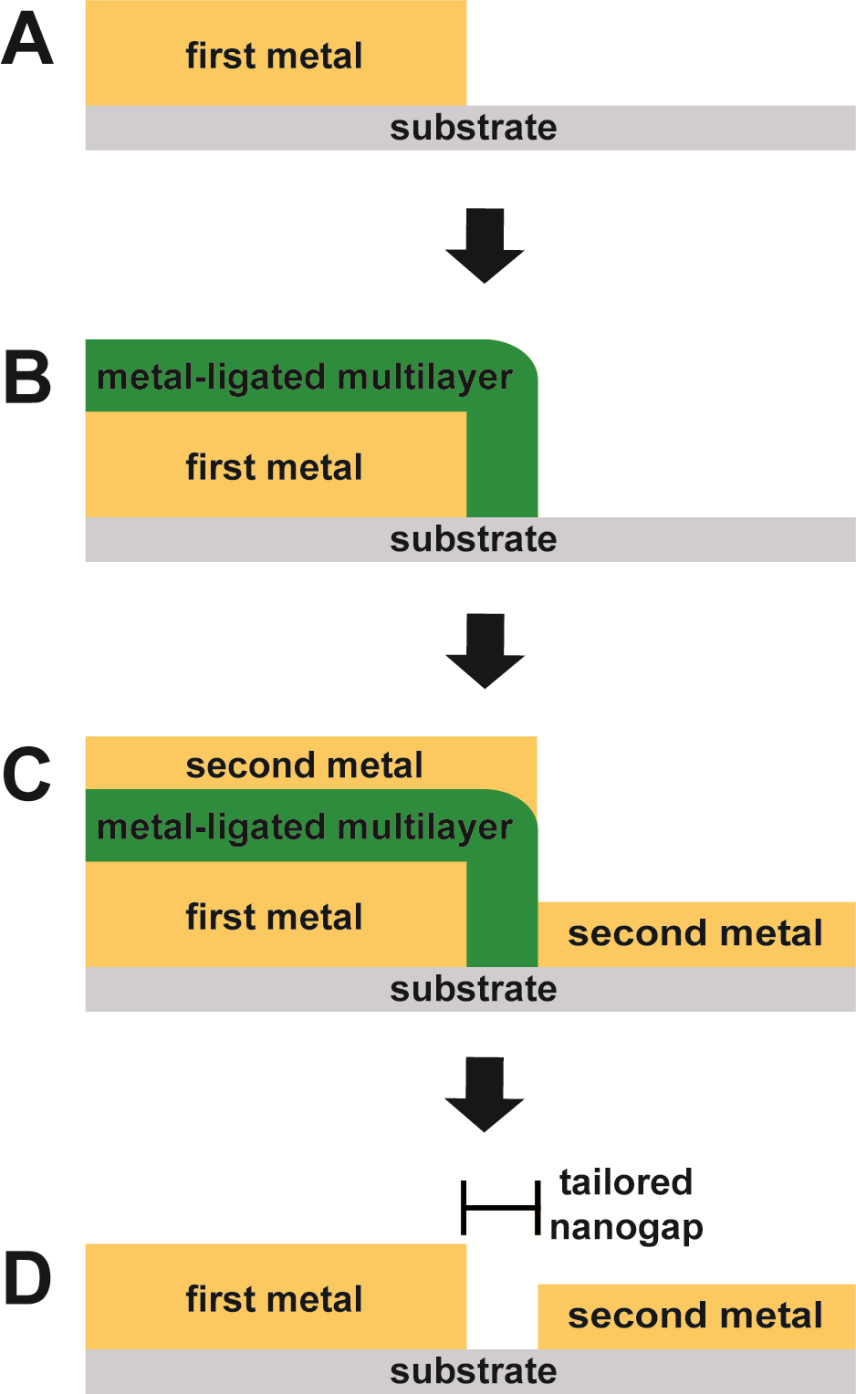
Key steps for the molecular-ruler process. (A) A metal is patterned on a substrate via conventional lithography. (B) A molecular-ruler, consisting of alternating layers of thiol molecules and metal ions, is created only on the first metal structure. (C) A second metal is deposited. (D) Upon removal of the molecular-ruler and the second metal on top of the multilayer via a chemical lift-off, a tailored nanogap is generated with a width that corresponds to the thickness of the multilayer.

The molecular-ruler process can be employed to form nanogaps between registered metal surface features that have been generated using conventional lithographic techniques such as photolithography or electron-beam lithography (Figure 1) [14–24]. In short, a metal structure that has been patterned on a non-metal substrate (e.g., Si) using conventional lithography is subsequently covered by a metal-ligated multilayer through the iterative deposition of bifunctional organic molecules and metal ions. Note that the use of a thiol as one of the two functionalities ensures that deposition and growth of the multilayer only occurs on the surface of the metal, not the exposed substrate. By using molecules of discrete length, the thickness of the multilayer can be precisely controlled through the number of deposition steps. Once the desired thickness has been achieved, a second metal deposition is used to cover the entire sample of the substrate, including the exposed substrate and the surface of the multilayer. Following this second metal deposition, a chemical lift-off removes the labile multilayer, thus exposing the initial metal feature and the portion of the substrate that was masked by the multilayer, yielding a nanogap between the two metal surfaces. The size of this gap is defined by the thickness of the multilayer. Utilization of the molecular-ruler process in this way provides a general and widely applicable method to fabricate registered, nanometer-scale features for potential applications including nanoelectronics, molecular-scale junctions, and electrochemical sensors [17–18,20–21,25–26].

**Figure 2:**
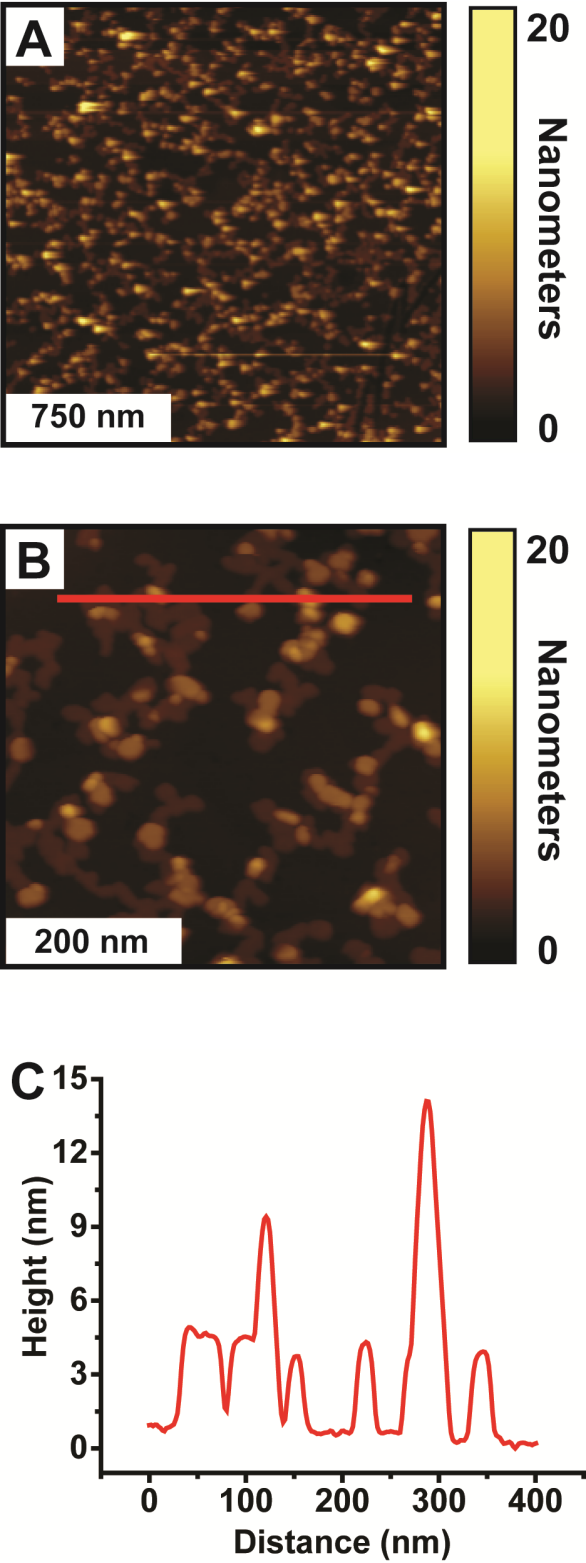
Cu-ligated MHDA-C16 bilayer formed from solution-phase deposition of C16. Representative (A) 2 µm × 2 µm and (B) 500 nm × 500 nm AFM images of a Cu-ligated MHDA-C16 bilayer formed from the solution deposition of MHDA for 18 h, Cu(ClO_4_)_2_·6H_2_O for 5 min, and C16 for 1 h. (C) Corresponding cursor profile across the C16 islands.

Although mercaptoalkanoic acid molecules, such as 16-mercaptohexadecanoic acid (MHDA), are the most widely studied molecules used in the molecular-ruler process, this method is inherently versatile through the use of molecules with alternate functionalities [27–31]. Towards this end, we set out to explore the use of an alkanethiol, specifically 1-hexadecanethiol (C16), as the terminating layer of a metal-ligated multilayer. This molecule was selected as it is commonly used to produce well-ordered self-assembled monolayers, has a relatively well understood terminal functionality (e.g., a methyl group), and enables direct comparison of thickness to MHDA molecules. Figures 2A and 2B show representative 2 µm × 2 µm and 500 nm × 500 nm atomic force microscopy (AFM) images of a Cu-ligated MHDA-C16 bilayer formed from the solution deposition of MHDA for 18 h, Cu(ClO_4_)_2_·6H_2_O for 5 min, and C16 for 1 h. Figure 2C displays a representative cursor profile across several islands as indicated by the red line in Figure 2B. Although C16 is very similar in structure to MHDA, the solution deposition of C16 results in structures that exhibit islands of various apparent heights, ranging from 3.4 to 24.8 nm, with relatively low surface coverages (38.2 ± 3.3%). This is in contrast to Cu-ligated MHDA bilayers, which exhibit islands of uniform height (ca. 2.2 nm) and have surface coverages of about 50% [27–29,32]. The C16 islands of the Cu-ligated MHDA-C16 bilayers are observed across the Au{111} substrate and are attributed to C16 molecules bound to a MHDA monolayer via cupric ions. The morphology of these islands is consistent with previous AFM topographic images of solution-deposited Cu-ligated MHDA-C16 bilayers [27]. This surface morphology results in a RMS roughness of 3.2 ± 0.5 nm, which is considerably larger than previously reported RMS roughnesses for MHDA monolayers (ca. 0.1 nm) and MHDA bilayers (1.0 nm) [32]. Similar morphology and slightly higher coverages of the C16 islands are observed when C16 is deposited from solution at 80 °C (Figure S1, Supporting Information File 1). Given the roughness and variations in the surface morphology of the Cu-ligated MHDA-C16 bilayers, it seems that the solution deposition of C16 is not suitable for use in the molecular-ruler process, and specifically for producing nanogaps with reproducible uniformity.

**Figure 3:**
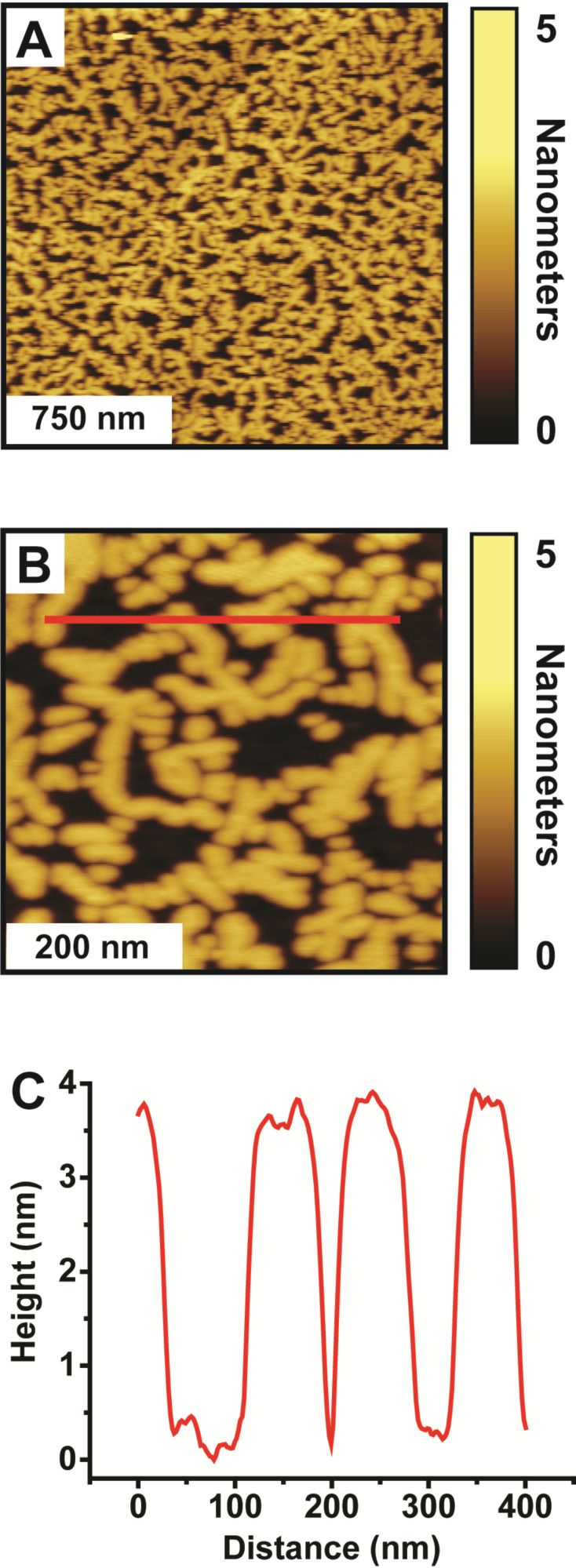
Cu-ligated MHDA-C16 bilayer formed from vapor-phase deposition of C16. Representative (A) 2 µm × 2 µm and (B) 500 nm × 500 nm AFM images of a of a Cu-ligated MHDA-C16 bilayer formed from the solution deposition of MHDA for 18 h and Cu(ClO_4_)_2_·6H_2_O for 5 min and the vapor deposition of C16 for 1 h at 80 °C. (C) Corresponding cursor profile across the C16 islands.

To overcome this limitation, the vapor deposition of C16 is explored. Interestingly, when C16 is deposited from the vapor phase onto MHDA monolayers to produce Cu-ligated MHDA-C16 bilayers, protruding islands with uniform thickness are observed across the Au{111} substrate (Figure 3). Figure 3A and Figure 3B show representative 2 µm × 2 µm and 500 nm × 500 nm AFM images of a Cu-ligated MHDA-C16 bilayer formed from the solution deposition of MHDA for 18 h and Cu(ClO_4_)_2_·6H_2_O for 5 min followed by vapor deposition of C16 for 1 h at 80 °C. Figure 3C displays a representative cursor profile across several islands as indicated by the red line in Figure 3B. The apparent height of these protruding islands (3.6 ± 0.2 nm) is consistent with the least-protruding C16 islands of the Cu-ligated MHDA-C16 bilayers formed via solution deposition. Protruding islands of greater thicknesses are not observed. The surface morphology of the Cu-ligated MHDA-C16 bilayer formed via vapor deposition results in a RMS roughness of 1.3 ± 0.1 nm, which is smaller than a Cu-ligated MHDA-C16 bilayer formed via solution deposition. Further, the surface coverage of these C16 islands (69.9 ± 1.8%) is considerably higher than the C16 surface coverage for the MHDA-C16 bilayer formed via solution deposition. Given the increase in surface coverage coupled with the marked decrease in roughness, this method is far more amendable to our goal of nanogap formation. It should be noted that thickness of the C16 islands is roughly twice as thick as predicted, which has been observed in other studies [27,33–34]. Although the explanation of this height discrepancy it not completely clear, it is conceivable that the doubling in height results from disulfides that are intercalated into the hydrocarbon tails of the Cu-ligated C16 molecules.

**Figure 4:**
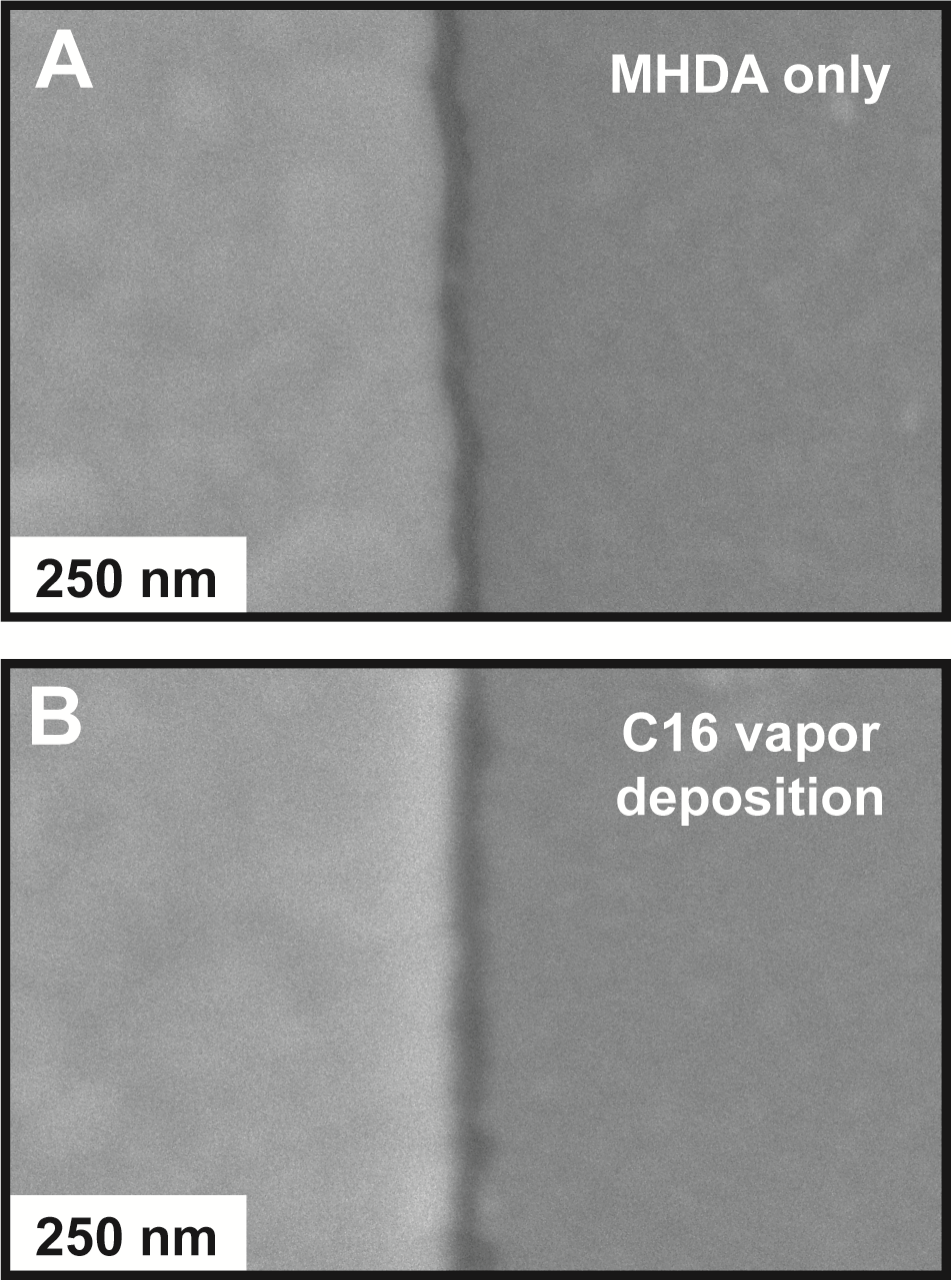
Nanogaps from MHDA only and MHDA with vapor-phase deposition of C16. (A) A representative SEM image of a nanogap fabricated from nine iterations of the solution deposition of MHDA and Cu(ClO_4_)_2_·6H_2_O followed by the solution deposition of MHDA. (B) A representative SEM image of a nanogap from ten iterations of the solution deposition of MHDA and Cu(ClO_4_)_2_·6H_2_O followed by the vapor deposition of C16. In both SEM images, the initial Au structure (100 nm thick) is on the left, and the second layer of Au (30 nm thick) is on the right.

To illustrate the applicability of the vapor-phase deposition of C16 in the molecular ruler process, Cu-ligated MHDA multilayers with and without a C16 capping layer are utilized to create nanogaps via the molecular-ruler process. Figure 4A shows a scanning electron microscope (SEM) image of the resulting nanogaps from nine iterations of the solution deposition of MHDA and Cu(ClO_4_)_2_·6H_2_O followed by the solution deposition of MHDA for 1 h. The higher-intensity region corresponds to the first Au deposition (100 nm thick) before multilayer growth, and the lower-intensity region corresponds to the second Au deposition (30 nm thick) after multilayer growth. The lowest-intensity region between the two Au regions corresponds to the nanogap where the Si substrate is exposed. This nanogap measures 26.0 ± 4.3 nm and is consistent with the thickness of the Cu-ligated MHDA decalayer measured via spectroscopic ellipsometry (24.8 ± 0.1 nm) and the thickness of Cu-ligated MHDA decalayers from previous studies [28].

Figure 4B shows an SEM image of the resulting nanogaps from ten iterations of the solution deposition of MHDA and Cu(ClO_4_)_2_·6H_2_O followed by the vapor deposition of C16 at 80 °C for 1 h. Similar higher and lower intensity regions are observed and correspond to the first and second Au deposition steps. The nanogap between the two Au regions measures 31.0 ± 9.4 nm, which is both larger and exhibits greater variability than the nanogap without the C16 capping layer. The width is consistent with the thickness of a Cu-ligated MHDA decalayer with the C16 capping (31.0 ± 1.0 nm) measured via spectroscopic ellipsometry.

The standard deviations of the nanogap widths, thus the quality of the nanogaps, result from the morphologies of the Cu-ligated multilayers of MHDA only and MHDA with vapor-phase deposition of C16 (Figure S2, Supporting Information File 1). The surface morphology of the 10-layer Cu-ligated MHDA multilayer with a C16 capping layer appears rougher with protruding islands with larger cross sections when compared to the 10-layer Cu-ligated MHDA multilayer without a C16 capping layer. Although the nanogaps produced from the Cu-ligated MHDA multilayer with a C16 capping layer have somewhat larger standard deviation, these nanogaps illustrate that alternate chemical functionalities can be utilized in the molecular-ruler process.

In conclusion, Cu-ligated MHDA-C16 bilayers formed from the solution and vapor deposition of C16 have been characterized with AFM revealing varied surface morphologies. The solution deposition of C16 results in structures that exhibit protruding islands of varying heights with relatively low surface coverages. These results agree with previous AFM topographic images of solution deposited Cu-ligated MHDA-C16 bilayers [27]. The vapor deposition of C16 produces protruding islands with uniform apparent heights and relatively high surface coverages. Given the increase in surface coverage coupled with the marked decrease in roughness for C16 islands formed from the vapor-phase deposition, Cu-ligated MHDA multilayers, without and with a vapor-phase deposited C16 capping layer, were utilized to create nanogaps between Au features using the molecular-ruler process. Although the quality of the nanogaps formed using the vapor-phase deposited C16 capping layer is diminished (i.e., the standard deviation is larger) when compared to MHDA multilayers, this is a minor tradeoff considering this approach enables the utilization of molecules with alternate functionalities beyond carboxylic acid into the molecular-ruler process. Efforts to explore the underling mechanism for the increased thickness of the C16 islands and to apply this strategy to other bifunctional thiol molecules are ongoing.

## Supporting Information

Supporting Information features additional AFM data and experimental details.

File 1 Additional experimental data.
