# Supplementary material for: Expanding the molecular-ruler process through vapor deposition of hexadecanethiol
Source: Beilstein J Nanotechnol. 2017 Nov 7;8:2339–44. doi: 10.3762/bjnano.8.233 (PMC5687049; doi:10.3762/bjnano.8.233)
Supplement: File 1 — Additional experimental data. [file Beilstein_J_Nanotechnol-08-2339-s001.pdf]

Supporting Information

for

Expanding the molecular-ruler process through vapor deposition of hexadecanethiol

Alexandra M. Patron[1], Timothy S. Hooker[2], Daniel F. Santavicca[2], Corey P. Causey*[,§,1] and Thomas J. Mullen*[,¶,1]

Address: [1]Department of Chemistry, University of North Florida, Jacksonville, FL 32224, USA and [2]Department of Physics, University of North Florida, Jacksonville, FL 32224, USA

Email: Corey P. Causey* - corey.causey@unf.edu; Thomas J. Mullen* - tj.mullen@unf.edu

[*]Corresponding author

[§]Phone: +1 904 620 2098; Fax: +1 904 620 3535

[¶]Phone: +1 904 620 1377; Fax: +1 904 620 3535

Additional AFM data

Figures S1 shows a representative 500 nm × 500 nm AFM image of a Cu-ligated MHDA-C16 bilayer formed from the solution deposition of MHDA for 18 h, $Cu(ClO_4)_2 \cdot 6H_2O$ for 5 min, and C16 for 1 h at 80°C. Islands of various apparent heights with relatively low surface coverages (46.8 ± 2.9%) are observed. This surface morphology results in a RMS roughness of 3.8 ± 0.2 nm.

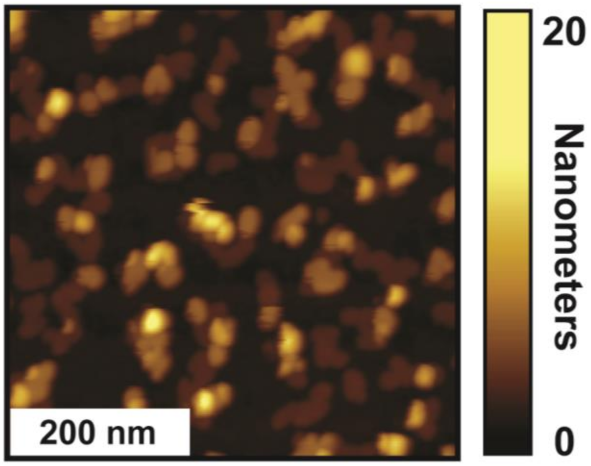

Figure S1: Cu-ligated MHDA-C16 bilayer formed from solution-phase deposition at 80 °C. Representative 500 nm × 500 nm AFM images of a Cu-ligated MHDA-C16 bilayer formed from the solution deposition of MHDA for 18 h, $Cu(ClO_4)_2 \cdot 6H_2O$ for 5 min, and C16 for 1 h at 80 °C.

Figure S2A shows a representative 500 nm × 500 nm AFM image of a Cu-ligated MHDA-C16 multilayer formed from nine iterations of the solution deposition of MHDA and $Cu(ClO_4)_2 \cdot 6H_2O$ followed by the solution deposition of MHDA for 1 h. Figure S2B displays a representative cursor profile as indicated by the red line in Figure S2A. The surface morphology results in a RMS roughness of 1.4 ± 0.1 nm. Additionally, isolated protruding features are observed across the substrate. These features have been observed previously and are attributed to the dimerization and agglomeration of MHDA molecules [1]. Figures S2C shows a representative 500 nm × 500 nm AFM image of a Cu-ligated MHDA multilayer formed from ten iterations of the solution

deposition of MHDA and $Cu(ClO_4)_2 \cdot 6H_2O$ followed by the vapor deposition of C16 at 80 °C for 1 h. Figure S2D displays a representative cursor profile as indicated by the red line in Figure S2C. The surface morphology results in a RMS roughness of 1.7 ± 0.1 nm. Isolated protruding features, with similar heights and larger cross sections when compared to the MHDA multilayers without the C16 capping layer, are observed across the substrate. These features are attributed to the C16 molecules depositing across the entire substrate including the dimerized and agglomerated MHDA molecules.

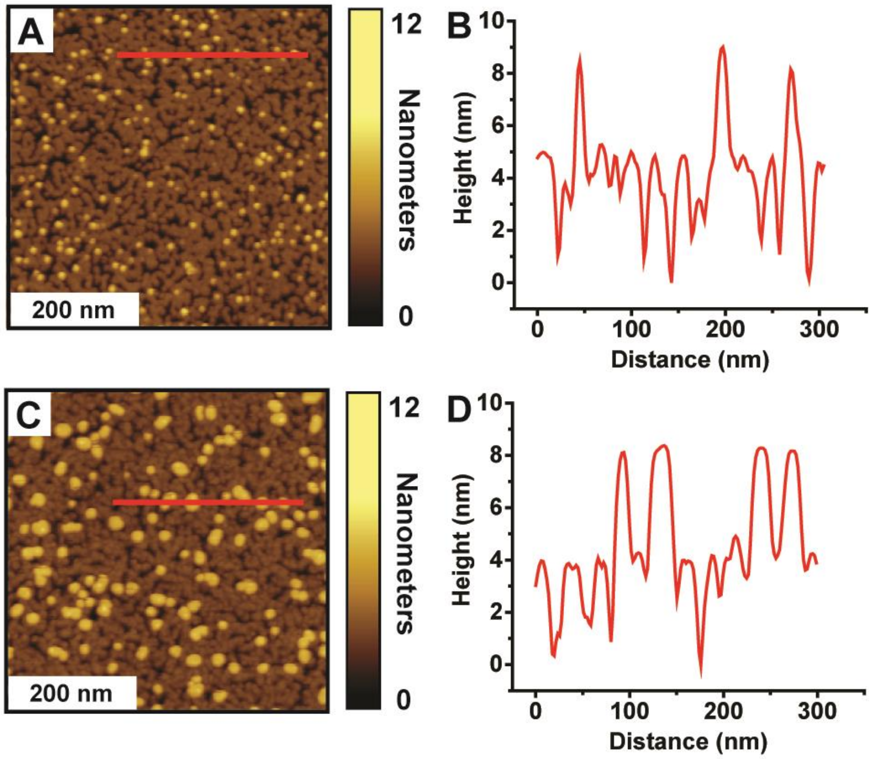

Figure S2: Cu-ligated MHDA multilayers from MHDA only and MHDA with vapor-phase deposition of C16. (A) A representative AFM image and (B) corresponding cursor profile of a Cu-ligated MHDA multilayer fabricated from nine iterations of the solution deposition of MHDA and $Cu(ClO_4)_2 \cdot 6H_2O$ followed by the solution deposition of MHDA for 1 h. (C) A representative AFM image and (D) corresponding cursor profile of a Cu-ligated MHDA multilayer from ten iterations of the solution deposition of MHDA and $Cu(ClO_4)_2 \cdot 6H_2O$ followed by the vapor deposition of C16 at 80 °C for 1 h.

Experimental details

Reagents and materials

16-Mercaptohexadecanoic acid (MHDA, 90%), 1-hexadecanethiol (C16, 95%), copper(II) perchlorate hexahydrate ($Cu(ClO_4)_2 \cdot 6H_2O$, 98%), and acetic acid (>99%) were purchased from Sigma-Aldrich (St. Louis, MO, USA). Nitric acid (ACS grade), hydrogen peroxide (30% aqueous solution), and sulfuric acid (ACS Grade) were purchased from VWR International (Randor, PA, USA). Absolute ethanol was purchased from Pharmco-Aaper (Bookfield, CT, USA). LOR-2A and Shipley 1813 photoresists, MF-319 developer, and Remover PG were purchased from MicroChem (Westborough, MA, USA). ACT-935 was purchased from Air Products (Allentown, PA, USA). All reagents were used as received. Water (18 MΩ) was generated using a Milli-Q system (Q-GARD 2, Millipore, Billerica, MA, USA). Au wire (99.99%, 1 mm diameter) was purchased from Scientific Instrument Services (Ringoes, NJ, USA). Pt–Ir foil (99.9%, 0.1 mm thick) was purchased from Alfa Aesar (Tewksbury, MA, USA). Undoped 2-inch silicon wafers were purchased from University Wafer (South Boston, MA, USA). All glassware was cleaned by immersing in piranha solution (3:1 by volume of sulfuric acid/30% hydrogen peroxide) for 1 h, rinsing with copious amounts of 18 MΩ water, and drying overnight in ambient. *Caution: piranha solution is a vigorous oxidant and should be used with extreme care!*

Preparation of Au substrates

For AFM experiments, Au beads with single crystal {111} facets were formed by melting Au wires mounted to Pt–Ir foils. Subsequently, the Au beads were positioned such that the single crystal {111} facets were parallel to the Pt–Ir foil [2-4]. The Au beads were cleaned by

immersing in hot (130 °C) nitric acid for 1 h, rinsed with copious amounts of 18 MΩ water, and dried under a stream of N_2. Au beads were annealed with a H_2 flame for 20–30 s just prior to immersion into thiol solutions.

For the spectroscopic ellipsometry and nanofabrication experiments, Au thin film structures were patterned on Si substrates using a bilayer resist process. A bilayer resist was employed to ensure a clean edge on the patterned Au structure. First, a 2″ Si wafer was spin-coated with LOR-2A photoresist and baked on a hotplate at 180 °C for 5 min. After cooling, it was spin-coated with Shipley 1813 photoresist and baked on a hotplate for 2 min. at 100 °C. Photolithographic patterning was performed using a Karl-Suss MA4 mask aligner with a UV exposure time of 25 s followed by submersion in MF-319 developer for 180 s. Following development, the wafer was rinsed in 18 MΩ water and dried under a stream of N_2. The wafer was then loaded into a thermal deposition system. After reaching a pressure of less than 10^{-6} Torr, 4 nm of Cr was deposited as an adhesion layer, followed by the deposition of 100 nm of Au. Lift-off was performed by soaking in Remover PG at 70 °C for 1 h followed by 5 min of sonication. The patterned wafers were then diced into four 1 cm × 3 cm chips. Each chip was cleaned by immersing in piranha solution for 30 min, rinsed with copious amounts of 18 MΩ water, dried under a stream of N_2. Subsequently, the chip was cleaned using a UV ozone cleaner (Novascan, PSDP-UVT, Ames, IA, USA) for 30 min, rinsed with absolute ethanol, and dried under a stream of N_2 prior to immersion into thiol solutions.

Preparation of Cu-ligated multilayers and nanogaps

Au substrates were immersed into 0.1 mM MHDA ethanoic solutions with 1.5 M acetic acid for 18–24 h. Acetic acid helps suppress the dimerization and agglomeration of the MHDA molecules

by competing for hydrogen-bonding interactions [1,5-7]. Subsequently, the Au substrates with the MHDA SAMs were rinsed with absolute ethanol, immersed into 5 mM $Cu(ClO_4)_2·6H_2O$ ethanolic solutions for 5 min, and rinsed with absolute ethanol. To grow multilayers of MHDA, the Au substrates were immersed into 1 mM ethanolic solutions of MHDA for 1 h and rinsed with absolute ethanol. This sequence of immersion into ethanolic solutions of $Cu(ClO_4)_2·6H_2O$ and MHDA was repeated until the number of desired layers was achieved. It is important to note that only the initial MHDA monolayer in Cu-ligated MHDA multilayers was assembled from a 0.1 mM MHDA ethanolic solution with 1.5 M acetic acid; the subsequent layers of the multilayers were assembled from 1 mM MHDA ethanolic solutions without acetic acid. We observed that 0.1 mM MHDA ethanolic solutions with 1.5 M acetic acid disrupt the assembly of the Cu-ligated MHDA multilayers and typically resulted in MHDA monolayers.

Two methods for the deposition, solution and vapor, of a terminal layer of C16 were explored. To deposit a C16 layer from solution, Au substrates with MHDA monolayers and/or multilayers were immersed into 5 mM $Cu(ClO_4)_2·6H_2O$ ethanolic solutions for 5 min, rinsed with absolute ethanol, and into 1 mM C16 ethanolic solutions for 1 h. The Au substrates were subsequently rinsed with absolute ethanol and dried under a stream of N_2. To deposit a C16 layer from vapor, Au substrates with MHDA monolayers and/or multilayers were immersed into 5 mM $Cu(ClO_4)_2·6H_2O$ ethanolic solutions for 5 min and rinsed with absolute ethanol. Approximately, 1 mL of neat C16 was pipetted into a 10 mL beaker. The Au substrates and the 10 mL beaker were then placed into a glass jar and sealed. The glass jar was held at 80 °C for 1 h, exposing the Au substrate to vapor-phase C16. The Au substrates were removed from the glass jar, rinsed with absolute ethanol, and dried under a stream of N_2. All SAMs and multilayers were imaged or utilized for hybrid-nanolithography immediately after preparation.

For the molecular-ruler process, samples were returned to the thermal deposition system. 3 nm of Cr was deposited followed by 30 nm of Au. Following deposition, the multilayer is removed by soaking the sample in ACT-935 at 75 °C for 1 h followed by 5 min of sonication. The sample is then rinsed in absolute ethanol and dried under a stream of N_2.

Atomic force microscopy

Tapping-mode (AC-mode) atomic force microscopy (AFM) images were acquired using an Agilent 5420 scanning probe microscope with OLTESPA Si cantilevers (Bruker AFM probes, Santa Barbra, CA, USA) with nominal force constants of 2 N/m. The Si cantilevers were cleaned using a UV/ozone cleaner (Novascan, PSDP-UVT, Ames, IA, USA) for 20 min to remove surface contaminants [8]. The drive frequency of the cantilever was offset by 0.1 kHz lower than the cantilever resonance to maintain repulsive probe-surface interactions [9,10]. The damping of the amplitude was set at 60–70% of free oscillation, and scan rates were set to 2.00 Hz. All AFM images were acquired at 256 points per line under ambient conditions. Image processing and analysis of the AFM images were performed using Gwyddion (version 2.48, "Magnetic Monastery"), which is an open-source software freely available on the internet and supported by the Czech Metrology Institute [11].

The AFM images used for analysis were acquired from several regions across each type of Cu-ligated multilayer, and the resulting apparent heights, surface coverages, and RMS roughnesses were averaged. The average and standard deviation (average ± standard deviation) of the apparent heights of the various features of the Cu-ligated multilayers were determined from cursor profiles from 500 nm × 500 nm AFM images across at least 30 features. The average and standard deviation (average ± standard deviation) of the surface coverages of the C16 islands

in the of Cu-ligated multilayers were calculated by counting the number of pixels above a thickness threshold and dividing by the total number of pixels in a set of at least four 500 nm × 500 nm AFM images for each type of multilayer. The thickness threshold values were determined by using the full-width-at-half-height across several protruding islands of C16 within the AFM images [12-18]. The average and standard deviation (average ± standard deviation) of the RMS roughnesses of the Cu-ligated multilayers were calculated using a set of at least four 500 nm × 500nm AFM images for each type of multilayer.

Spectroscopic ellipsometry

Spectroscopic ellipsometry measurements were acquired using a rotating compensator spectroscopic ellipsometer (Alpha-SE, J.A. Woollam Inc.) where 180 wavelengths between 380–900 nm were measured at a fixed 70° angle of incidence The Cu-ligated multilayer thicknesses were calculated using the CompleteEASE software package; a B-spline model was used for the Au substrate, and a Cauchy model was used for the multilayer film using a refractive index value of n = 1.5 [19-21]. Measurements were collected on multiple regions across each type of Cu-ligated multilayer, and the average and standard deviation (average ± standard deviation) of the resulting calculated thicknesses were determined.

Scanning electron microscopy

Scanning electron microscopy (SEM) images of the molecular-ruler samples were acquired using a TESCAN MIRA field-emission SEM. The size of the nanogap was determined by taking approximately 10 images at a magnification of 2×10^5 at various locations on each sample. On each image, the pixel intensity as a function of position was plotted for multiple linescans taken

perpendicular to the nanogap. A numerical procedure developed in the software program Igor Pro was used to determine the gap width by finding the full-width-at-half-minimum of each linescan. A total of 100 linescans for each sample (both without and with the C16 capping layer) were used to calculate the average and standard deviation (average ± standard deviation) of the nanogap width.

References

1. Drexler, C. I.; Moore, K. B.; Causey, C. P.; Mullen, T. J. *Langmuir* **2014,** *30*, 7447-7455.

2. Shao, J.; Josephs, E. A.; Lee, C.; Lopez, A.; Ye, T. *ACS Nano* **2013,** *7*, 5421-5429.

3. Josephs, E. A.; Ye, T. *J. Am. Chem. Soc.* **2012,** *134*, 10021-10030.

4. Calavilier, J.; Faure, R.; Guinet, G.; Durand, R. *J. Electroanal. Chem.* **1980,** *107*, 205-209.

5. Lee, C.; Josephs, E. A.; Shao, J.; Ye, T. *J. Phys. Chem. C* **2012,** *116*, 17625-17632.

6. Snow, A. W.; Jernigan, G. G.; Ancona, M. G. *Analyst* **2011,** *136*, 4935-4949.

7. Kelley, A. T.; Ngunjiri, J. N.; Serem, W. K.; Lawrence, S. O.; Yu, J.-J.; Crowe, W. E.; Garno, J. C. *Langmuir* **2010,** *26*, 3040-3049.

8. Lo, Y. S.; Huefner, N. D.; Chan, W. S.; Dryden, P.; Hagenhoff, B.; Beebe, T. P. *Langmuir* **1999,** *15*, 6522-6526.

9. Garcia, R.; Perez, R. *Surf. Sci. Rep.* **2002,** *47*, 197-301.

10. Weisenhorn, A. L.; Maivald, P.; Butt, H. J.; Hansma, P. K. *Phys. Rev. B* **1992,** *45*, 11226-11232.

11. Necas, D.; Klapetek, P. *Cent. Eur. J. Phys.* **2012,** *10*, 181-188.

12. Drexler, C. I.; Causey, C. P.; Mullen, T. J. *Scanning* **2015,** *37*, 6-16.

13. Brownfield, A. L.; Causey, C. P.; Mullen, T. J. *Thin Solid Films* **2015,** *594*, 184-191.

14. Brownfield, A. L.; Causey, C. P.; Mullen, T. J. *J. Phys. Chem. C* **2015,** *119*, 12455-12463.

15. Bu, D. L.; Mullen, T. J.; Liu, G. Y. *ACS Nano* **2010,** *4*, 6863-6873.

16. Dameron, A. A.; Hampton, J. R.; Smith, R. K.; Mullen, T. J.; Gillmor, S. D.; Weiss, P. S. *Nano Lett.* **2005,** *5*, 1834-1837.

17. Mullen, T. J.; Dameron, A. A.; Saavedra, H. M.; Williams, M. E.; Weiss, P. S. *J. Phys. Chem. C* **2007,** *111*, 6740-6746.

18. Mullen, T. J.; Srinivasan, C.; Hohman, J. N.; Gillmor, S. D.; Shuster, M. J.; Horn, M. W.; Andrews, A. M.; Weiss, P. S. *Appl. Phys. Lett.* **2007,** *90*.

19. Evans, S. D.; Ulman, A.; Goppertberarducci, K. E.; Gerenser, L. J. *J. Am. Chem. Soc.* **1991,** *113*, 5866-5868.

20. Benson, A. S.; Elinski, M. B.; Ohnsorg, M. L.; Beaudoin, C. K.; Alexander, K. A.; Peaslee, G. F.; DeYoung, P. A.; Anderson, M. E. *Thin Solid Films* **2015,** *590*, 103-110.

21. Daniel, T. A.; Uppili, S.; McCarty, G.; Allara, D. L. *Langmuir* **2007,** *23*, 638-648.
